# Novel Segment- and Host-Specific Patterns of Enteroaggregative *Escherichia coli* Adherence to Human Intestinal Enteroids

**DOI:** 10.1128/mBio.02419-17

**Published:** 2018-02-20

**Authors:** Anubama Rajan, Lucy Vela, Xi-Lei Zeng, Xiaomin Yu, Noah Shroyer, Sarah E. Blutt, Nina M. Poole, Lily G. Carlin, James P. Nataro, Mary K. Estes, Pablo C. Okhuysen, Anthony W. Maresso

**Affiliations:** a Department of Molecular Virology and Microbiology, Baylor College of Medicine, Houston, Texas, USA; b Department of Natural Sciences, University of Houston—Downtown, Houston, Texas, USA; c Department of Medicine Section of Gastroenterology and Hepatology, Baylor College of Medicine, Houston, Texas, USA; d Department of Pediatrics, University of Virginia School of Medicine, Charlottesville, Virginia, USA; e Department of Infectious Diseases, University of Texas MD Anderson Cancer Center, Houston, Texas, USA; UT Southwestern Med Center Dallas

**Keywords:** enteroaggregative *E. coli*, adherence, enteroid, fimbriae, intestine, tropism

## Abstract

Enteroaggregative *Escherichia coli* (EAEC) is an important diarrheal pathogen and a cause of both acute and chronic diarrhea. It is a common cause of pediatric bacterial diarrhea in developing countries. Despite its discovery in 1987, the intestinal tropism of the pathogen remains unknown. Cell lines used to study EAEC adherence include the HEp-2, T-84, and Caco-2 lines, but they exhibit abnormal metabolism and large variations in gene expression. Animal models either do not faithfully manifest human clinical symptoms or are cumbersome and expensive. Using human intestinal enteroids derived from all four segments of the human intestine, we find that EAEC demonstrates aggregative adherence to duodenal and ileal enteroids, with donor-driven differences driving a sheet-like and layered pattern. This contrasts with the colon, where segment-specific tropisms yielded a mesh-like adherence pattern dominated by interconnecting filaments. Very little to no aggregative adherence to jejunal enteroids was observed, regardless of the strain or donor, in contrast to a strong duodenal association across all donors and strains. These unique patterns of intestinal segment- or donor-specific adherence, but not the overall numbers of associated bacteria, were dependent on the major subunit protein of aggregative adherence fimbriae II (AafA), implying that the morphology of adherent clusters and the overall intestinal cell association of EAEC occur by different mechanisms. Our results suggest that we must give serious consideration to inter- and intrapatient variations in what is arguably the first step in pathogenesis, that of adherence, when considering the clinical manifestation of these infections.

## IMPORTANCE

EAEC is a leading cause of pediatric bacterial diarrhea and a common cause of diarrhea among travelers and immunocompromised individuals. Heterogeneity in EAEC strains and lack of a good model system are major roadblocks to the understanding of its pathogenesis. Utilizing human intestinal enteroids to study the adherence of EAEC, we demonstrate that unique patterns of adherence are largely driven by unidentified factors present in different intestinal segments and from different donors. These patterns are also dependent on aggregative adherence fimbriae II encoded by EAEC. These results imply that we must also consider the contribution of the host to understand the pathogenesis of EAEC-induced inflammation and diarrhea.

## INTRODUCTION

Enteroaggregative *Escherichia coli* (EAEC) is a heterogeneous group of enteric bacteria that is a major cause of acute and persistent diarrhea, illness, and death among children in developing countries (1). Chronic infection of young children may lead to malnourishment and cognitive impairment (2). Infection becomes chronic in immunocompromised patients and is the second most common cause of traveler’s diarrhea (3–6). A deadly outbreak of EAEC O104:H4 in Germany in 2011 involved >4,000 Europeans, with at least 50 deaths (7). In addition, EAEC is commonly associated with diarrheal illness in inpatient and emergency units in the United States and >65% of EAEC isolates are multidrug resistant (8–11). The clinical symptoms of EAEC infection include watery or bloody diarrhea and sometimes fever and mucoid stools (12–14). In some patients, the disease is acute, lasting only a few days; in others, it is persistent and can last >2 weeks (mean) (12). Elevated levels of inflammatory markers in stool, including interleukin-8 and lactoferrin, have been reported, but this is not universally observed (2).

The observation by James Nataro and Myron Levine of a unique “aggregative” adherence of *E. coli* isolated from Chilean children presenting with diarrhea that forms a “stacked-brick” structure on HEp2 cells led to the discovery of a new pathotype named enteroaggregative *E. coli* or EAEC (15). Since this observation, numerous epidemiological and human volunteer studies have cemented EAEC as a substantial cause of human diarrhea (16). Adherence is often the defining physical feature that differentiates the various *E. coli* pathotypes from each other and therefore is often considered an important first step in the pathogenesis of diarrhea (17). The adhesive properties of EAEC are largely dictated by four types of aggregative adherence fimbriae (AAFs), AggA (AAF/I), AafA (AAF/II), Agg3A (AAF/III), and Agg4A (AAF/IV), all encoded by the plasmid of aggregative adherence (pAA) and regulated positively by the transcriptional activator AggR; a fifth adhesin, Agg5A (AAF/V), was recently discovered (18–25). Additionally, in some strains, aggregative adherence may also be mediated by outer membrane proteins (26, 27). In addition to adherence factors, pAA also encodes cytoactive effectors or toxins, including heat-stable toxin 1 (EAST-1, which activates cAMP) (28), plasmid-encoded toxin (Pet, which degrades the host cytoskeleton) (29), and serine protease autotransporters (SPATES) such as Pic (a mucinase) (29, 30). An antiaggregation protein termed dispersin (31), as well as the dispersin transporter (32), has been described. On the basis of the presence of AggR or pAA, EAEC strains are classified as typical (AggR^+^) or atypical (AggR^−^) (17, 33–36). Despite the elegant work that has elucidated the molecular function of these bacterial factors, it is still uncertain whether they define a tropism for one portion of the gastrointestinal tract or another and whether this tropism can be responsible for the divergent range of symptoms and susceptibility to and duration of disease in those who are infected.

Enteroids or “miniguts” are recently developed organotypic culture systems derived from crypts isolated from human intestinal biopsy specimens (37–39). They are stimulated with the growth factors Wnt, Noggin, and R-spondin to retain structural features of a miniature gut (lumen, villi, and crypts) (37, 40–42), are heterocellular containing multiple epithelial cell types (enterocytes and goblet, enteroendocrine, and Paneth cells), and can be grown either as a three-dimensional (3D) organotypic system or as monolayers (43–47). In this study, we investigated the adherence of EAEC to human intestinal enteroids with the hope of determining the host contribution to what is arguably the first step in the pathogenesis of bacteria that cause human diarrhea.

## RESULTS

**FIG 1:**
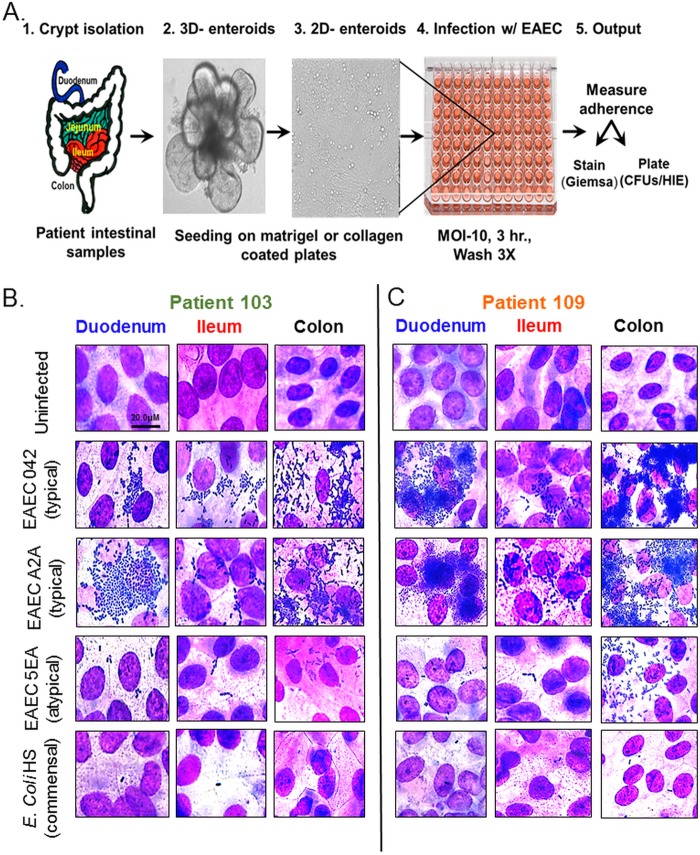
Patterns of EAEC adherence to HIEMs obtained from different donors and segments of the intestine. (A) Schematic representation of the methods used to assess adherence to enteroid monolayers. (B and C) Duodenal, ileal, and colon differentiated 2D HIEMs obtained from donors 103 and 109, respectively, were infected with EAEC 042 (a prototype strain), EAEC A2A (clinical isolate, typical EAEC), EAEC 5EA (clinical isolate, atypical EAEC), and *E. coli* HS (nonpathogenic control strain) at an MOI of 10 for 3 h. After infection, the cells were washed, fixed, stained with Giemsa-Wright stain, and imaged at ×100 to visualize the pattern of bacterial adherence. Technical replicates, three wells representative of 12 images; six biological replicates (two donors and three segments).

We made cultures from crypts isolated from tissues from four different segments of the intestines of donors who underwent biopsy or bariatric surgery at the Texas Medical Center (Fig. 1A). Crypts were cultured in Matrigel in proliferation medium and expanded into 3D enteroids over 7 days by the methods developed by Sato and Cleavers, and two-dimensional (2D) enteroid monolayers were ultimately prepared on Matrigel or collagen-coated plates from established cultures by the methods of Van Dussen et al. (37, 40, 47, 48). Reasoning that some of the pleiotropic effects upon infection with this pathogen may be due to an intestinal segment tropism, we characterized the overall and type of adherence of EAEC to three different intestinal segments from the same donor. Duodenal, ileal, and colonic enteroids became 100% confluent after 4 days of differentiation, as assessed by Giemsa-Wright staining (Fig. 1B). When *E. coli* HS, a nonpathogenic, non-diarrhea-causing strain, was added to each culture, very little, if any, adherence was observed (Fig. 1B). When EAEC strain 042, a clinical isolate that caused diarrheal disease in human challenge studies and demonstrates aggregative adherence on HEp2 cells (so-called stacked-brick adherence), was added to these three cultures, distinct patterns of adherence were observed. The classic stacked-brick aggregative adherence pattern, the phenotype that has defined this pathogen, was observed in the duodenum and ileum. Adherence to colon enteroids, however, was unique and can be characterized as a mesh-like pattern with thin filaments connecting clusters of three to five bacteria. When this experiment was repeated with cultures from a different donor (no. 109, Fig. 1C), the stacked-brick phenotype was again observed in the ileum and the mesh-like pattern was observed in the colon, similar to donor 103. Unexpectedly, EAEC 042 added to the duodenum demonstrated large clusters of multilayered bacterial groups, a result that was in stark contrast to that observed in donor 103.

The finding that the same bacterial strain demonstrated two distinct adherence patterns on cultures from different tissues from the same donor, as well as cultures from the same tissues from different donors, suggests that the host is also a driver of adherence. To further test this hypothesis, we isolated from a donor with diarrhea another strain of EAEC that was positive by PCR for *aggR* (the master regulator of adherence) and shows aggregative adherence to Hep2 cells (Table 1). This strain (A2A) was added to enteroid monolayers derived from donors 103 and 109, and the adherence pattern was recorded. Like EAEC 042, strain A2A demonstrated a stacked-brick pattern of adherence to the ileum and a mesh-like pattern of adherence to the colon (Fig. 1C) in donors 103 and 109. Whereas these two patterns were observed in the ileum and colon, respectively, the layered grouping of bacteria was only discernible in the duodenum of donor 109, where large clusters of EAEC cells formed in “balls” of bacteria that numbered several hundred in a single grouping. In contrast, no balls were ever observed in the duodenum of donor 103. Instead, a sheet-like arrangement of grouped bacteria one cell thick was observed (Fig. 1B). These patterns of adherence were also observed in duodenum and ileum cultures from a third donor (see Fig. S1 in the supplemental material). In fact, the infected duodenal cultures from three different donors also showed the ball and sheet-like patterns, while the stacked-brick pattern was retained by three different ileal segment cultures (Fig. S4). Of potential significance, when the adherence pattern of EAEC 042 was assessed with jejunal enteroids taken from three different donors, very little, if any, adherence was observed (Fig. S2). In fact, the weak adherence resembled that of *E. coli* HS. None of these adherence patterns were observed in another clinical isolate of EAEC that lacks *aggR* and is considered atypical (strain 5EA, Table 1), regardless of the segment or donor used (Fig. 1B and C).

10.1128/mBio.02419-17.1 FIG S1  Patterns of EAEC adherence to a third donor, 104. Duodenal and ileal 2D differentiated HIEMs obtained from donor 104 were infected with EAEC 042, EAEC A2A, EAEC 5EA, and *E. coli* HS at an MOI of 10 for 3 h. The cells were then washed, fixed, stained with Giemsa-Wright stain, and imaged at ×100 to visualize the pattern of adherence. Technical replicates, three wells representative of 12 images; two biological replicates (two segments from one donor). Download FIG S1, TIF file, 0.5 MB. Copyright © 2018 Rajan et al. 2018 Rajan et al. This content is distributed under the terms of the Creative Commons Attribution 4.0 International license.

10.1128/mBio.02419-17.2 FIG S2  Adherence of EAEC to jejunal HIEMs. (A) 2D jejunal HIEMs obtained from three different donors, J2, J3, and J11, were infected with EAEC 042, EAEC A2A, and EAEC 5EA at an MOI of 10 for 3 h. The cells were then washed, fixed, stained with Giemsa-Wright stain, and imaged at ×100 to visualize the pattern of adherence. (B) The total level of adhered EAEC from infections identical to those performed as described for panel A. Data represent the mean values of three independent experiments, and the error bars denote the standard error of the mean. Technical replicates, three wells representative of 12 images; three biological replicates (three enteroid lines obtained from different donors) Download FIG S2, TIF file, 0.5 MB. Copyright © 2018 Rajan et al. 2018 Rajan et al. This content is distributed under the terms of the Creative Commons Attribution 4.0 International license.

**TABLE 1:** Details of the EAEC strains used in this study

No.	Strain	Characteristic	Adherence pattern	Presence of:					Source^a^
				AggR	AatA	Aap	Pic	Pet	
1	EAEC 042	Prototype strain expressing AAF/II (*aafA*)	Aggregative	+	+	+	+	+	P.C.O.
2	EAEC A2A	Clinical isolate expressing AAF/II (*aafA*)	Aggregative	+	+	+	+	+	P.C.O.
3	EAEC 5EA	Clinical isolate expressing AAf/III (*agg3* cluster)	Diffuse	−	−	−	+	−	P.C.O.
4	JM221	EAEC strain expressing AAF/I (*aggA* cluster)	Aggregative	+	+	+	+	−	J.N.
5	*E. coli* HS	Lab-adapted commensal	None	−	−	−	−	−	P.C.O.
6	EAEC 042 Δ*aafA*	*aafA*, a major fimbrial subunit of AAF/II mutant	Diffuse	+	+	+	+	+	J.N.
7	EAEC 042 Δ*aggR*	*aggR*, master regulator of pAA mutant	Diffuse	−	+	+	+	+	J.N.

a P.C.O., Pablo C. Okhuysen; JN, James Nataro.

**FIG 2:**
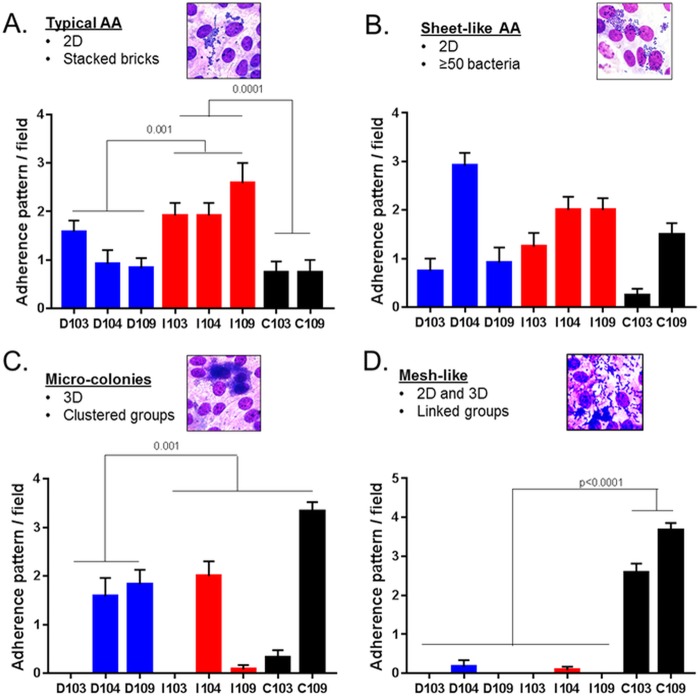
Definition and quantification of the different patterns of EAEC aggregative adherence on differentiated 2D HIEMs. (A to D) The insets show the occurrence of typical, sheet-like, microcolonies and mesh-like aggregative adherence (AA) exhibited by EAEC 042 across different donors and segments of intestine during infection as described in the legend to Fig. 1. Samples (D, duodenum [blue]; I, ileum [red]; C, colon [black]) were obtained from donors 103, 104, and 109, and the adherence pattern was quantified from an average of 12 different fields of 0.8 mm^2^ taken at ×100. Data represent the mean values of three independent experiments, and the error bars denote the standard error of the mean. Technical replicates, three wells representative of 12 images; eight biological replicates (three donors and three segments).

These qualitative findings indicate that there may be a segment- and donor-specific adherence tropism for the interaction of EAEC with human enteroids. To investigate this further, we quantified these various adherence patterns by defining their features. Typical stacked-brick adherence was defined as having a 2D array with clusters of 15 to 50 bacteria (Fig. 2A, inset). Sheet-like adherence also contains a 2D array, but the stacked-brick cluster occurs in large sheets of >50 bacteria (Fig. 2B, inset). Microcolony-like adherence consists of large balls of three-dimensionally arrayed bacteria that contain, by our best estimates, >200 bacteria per ball (Fig. 2C, inset). Mesh-like adherence displays features of both sheet-like and layered adherence, with many clusters of three to five bacteria linked in a mesh-like array (Fig. 2D, inset). These clusters are seemingly linked by thin filaments that extend over each cluster (Fig. S3).

10.1128/mBio.02419-17.3 FIG S3  Mesh-like adherence to colon cell monolayers. Pattern of EAEC adherence to colon monolayers demonstrates a unique mesh-like pattern, where groups of aggregative adhering bacteria are connected to each other through chains or strings of bacteria. The bottom images show enlarged examples of this property. Download FIG S3, TIF file, 1 MB. Copyright © 2018 Rajan et al. 2018 Rajan et al. This content is distributed under the terms of the Creative Commons Attribution 4.0 International license.

10.1128/mBio.02419-17.4 FIG S4  Pattern of EAEC 042 adherence to enteroid monolayers derived from duodenum and ileum samples from different donors. Duodenal and ileal HIEMs obtained from samples 4D, 8D, D112, I11, I12, and I16 were infected with EAEC 042 at an MOI of 10 for 3 h. After infection, the cells were washed, fixed, stained with Giemsa-Wright stain, and imaged at ×100 to visualize the pattern of bacterial adherence. Download FIG S4, TIF file, 0.4 MB. Copyright © 2018 Rajan et al. 2018 Rajan et al. This content is distributed under the terms of the Creative Commons Attribution 4.0 International license.

When scored in accordance with these parameters, the duodenum and ileum demonstrated clear brick- and sheet-like adherence patterns, unlike the jejunum and colon (Fig. 2A and B). Microcolonies were seen predominantly in the duodenal segment of the intestine rather than in the ileum or colon (Fig. 2C). Donors 104 and 109, but not donor 103, also demonstrated layered aggregated adherence (Fig. 2B). Of the four parameters assessed (typical stacked-brick, microcolony, sheet-like, and mesh-like patterns), only the colon demonstrated the mesh-like pattern (Fig. 2D), especially that of donor 103, which showed infrequent layered adherence (Fig. 2B).

**FIG 3:**
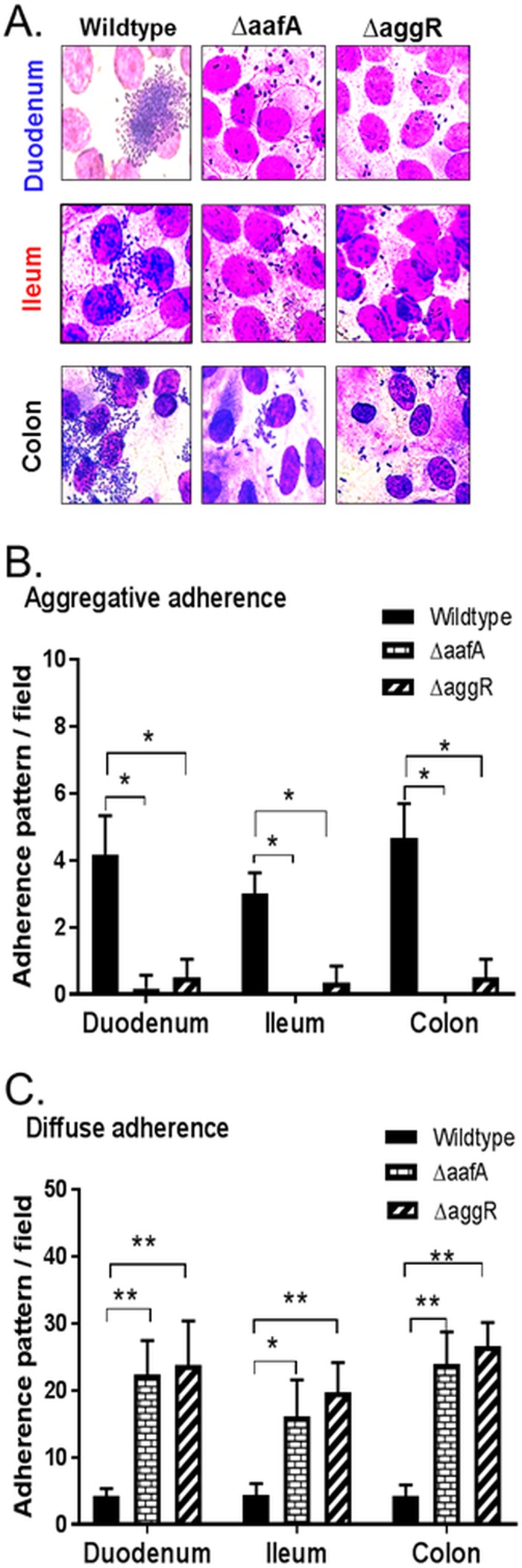
Adherence of wild-type EAEC (042) and Δ*aafA* and Δ*aggR* mutant strains to 2D differentiated HIEMs. (A) Wild-type EAEC and Δ*aafA* and Δ*aggR* mutants were added to duodenal, ileal, or colonic enteroids obtained from donor 103 as described in the legend to Fig. 1, and the adherence pattern was visualized by Giemsa-Wright staining. (B and C) The four adherence patterns from Fig. 2, classified collectively as aggregative adherence, were quantified versus that of diffuse adherence (see Materials and Methods). Data represent the mean values of three independent experiments, and the error bars denote the standard error of the mean, *, *P* < 0.05; **, *P* < 0.01. Technical replicates, three wells representative of 12 images; three biological replicates (one donor and three segments).

The finding that strain 5EA, which was negative for *aggR* by PCR and did not form any of the above adherence patterns (but rather adhered diffusely) implies that adherence to enteroids depends on the so-called aggregated adherence fimbriae, which are regulated by *aggR*. To test this hypothesis, we used strains of EAEC 042 harboring isogenic deletions in *aafA* (the predominant fimbriae responsible for stacked-brick adherence to HEp2 cells) and *aggR* (the positive regulator of aggregative adherence) (21, 49, 50). As shown in Fig. 3A, wild-type EAEC 042 demonstrated microcolony adherence to the duodenum, typical stacked-brick adherence to the ileum, and mesh-like adherence to colon enteroids from donor 103, as observed previously. EAEC strain 42, without *aafA* or *aggR*, did not show any of these adherence patterns; instead, sporadic clusters of two or three bacteria diffusely spread out over the monolayers of all segments were observed (Fig. 3A). When quantified for the stacked-brick, layered, and mesh-like aggregated adherence subtypes, only the wild-type 042 strain demonstrated this pattern relative to the strains lacking *aafA* or *aggR* (Fig. 3B). In fact, when quantified for diffuse adherence (defined as clusters of one to four bacteria), the *aafA*- and *aggR*-deficient strains demonstrated this phenotype much more prominently than the wild-type strain (Fig. 3C). In support of these findings, an EAEC strain we recently isolated from a donor that naturally lacks AAF/II (in fact, it only has AAF/I; Table 1) also demonstrated diffuse adherence on enteroids (Fig. S5).

10.1128/mBio.02419-17.5 FIG S5  Pattern of EAEC strain JM221 adherence to enteroid monolayers. EAEC strain JM22, which expresses AAF/I, was added to enteroid monolayers (MOI of 10, 3 h) derived from duodenal (D103), jejunal (J3), and ileal (I11) segments. After infection, cells were washed, fixed, stained with Giemsa-Wright stain, and imaged at ×100 to visualize pattern of bacterial adherence. Download FIG S5, TIF file, 0.1 MB. Copyright © 2018 Rajan et al. 2018 Rajan et al. This content is distributed under the terms of the Creative Commons Attribution 4.0 International license.

**FIG 4:**
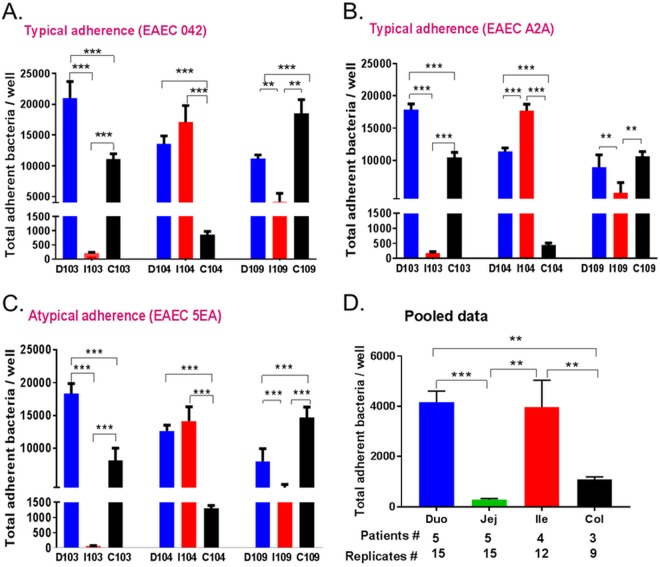
Total adherence of EAEC to three intestinal segments from three different donors. (A to C) 2D differentiated HIEMs obtained from duodenal, ileal, and colonic segments from donors 103, 104, and 109 were infected with EAEC 042 (A), A2A (B), or 5EA (C) at an MOI of 10 for 3 h. (D) Pooled adherence data from enteroids from all four intestinal segments from additional donors. Adherence was quantified as described in Materials and Methods. Data represent the mean values of three independent experiments, and the error bars denote the standard error of the mean. **, *P* < 0.01; ***, *P* < 0.001. Technical replicates, three wells; 9 (three donors and three segments) (A to C) and 17 (D) biological replicates.

The distinct patterning observed in enteroids derived from different segments of the intestine, along with the donor-specific patterning, implies that an unidentified adherence mechanism is at play in EAEC in the human intestine. We next wondered if this was also true of the total level of adherence to enteroid monolayers. Using enteroids from three different segments and from three different donors, we examined the total number of EAEC bacteria that adhered after a 3-h incubation. When examined in this manner, donor 103 showed high EAEC adherence to the duodenum (Fig. 4A, blue). This was in contrast to the observed adherence to cultures from the ileum, which was very low, and the colon, which was intermediate between the two. Confidence in the rigor of this observation was confirmed by the use of EAEC A2A, which demonstrated a total adherence outcome nearly identical to that of donor 103 (Fig. 4B). Strikingly, despite its overall diffuse adherence (no stacked-brick, layered, or mesh-like pattern), which was attributed to the loss of *aggR* (Fig. 3), EAEC strain 5EA demonstrated similar up-down-intermediate binding to the duodenum, ileum, and colon enteroids of donor 103, respectively (Fig. 4C). When this experiment was repeated with enteroids from donor 104, a different total adherence was observed. Whereas the duodenum retained high levels of adherence, the levels of adherence to the ileum were also high (in some cases exceeding adherence to the duodenum across all three EAEC strains [Fig. 4A to C, donor 104]). The colon, however, showed less overall adherence by all three strains, and the adherence was also lower than in the colon of donor 103. In contrast to donors 103 and 104, donor 109 demonstrated strong total adherence to all three segments of the intestine by all three EAEC strains, with the colon demonstrating the highest overall level of binding (Fig. 4A to C). In another set of experiments, we pooled the adherence results of strain 42 from enteroids from different segments from multiple donors in our enteroid bank. As shown in Fig. 4D, the duodenum demonstrated the highest level of adherence, followed by the ileum and colon. As observed throughout this study, low levels of total adherence were observed in the jejunum.

**FIG 5:**
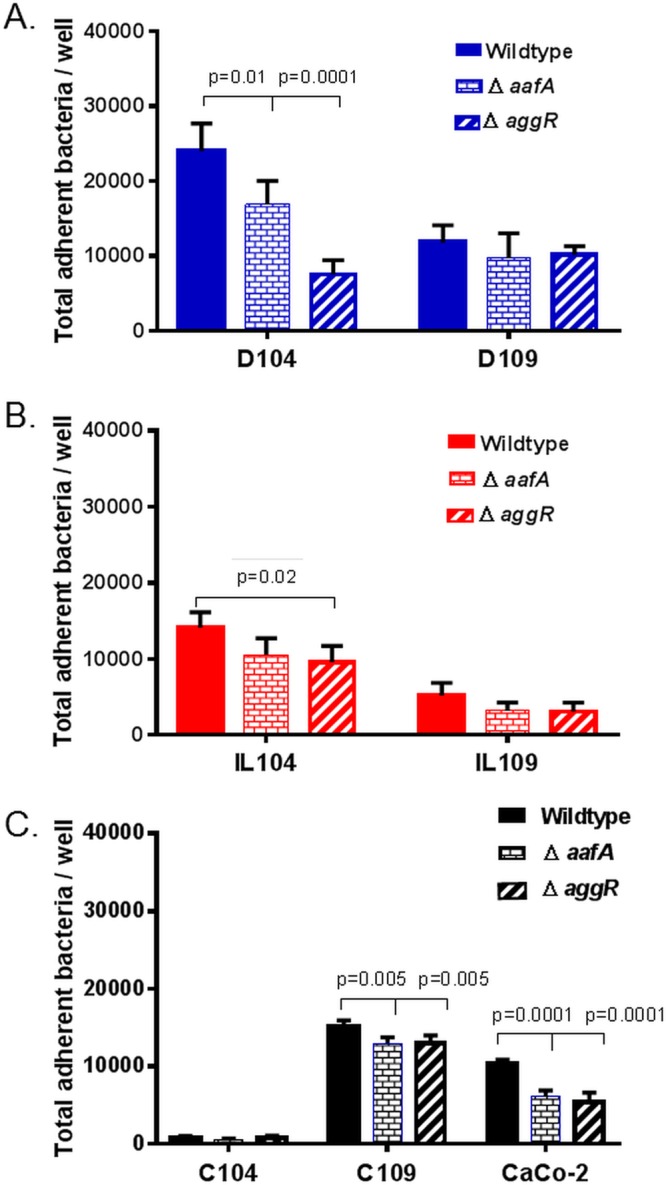
Total adherence of wild-type and mutant EAEC to HIEMs. (A, B) Wild-type EAEC and Δ*aafA* and Δ*aggR* mutants were incubated with 2D differentiated HIEMs from duodenum, ileum, and colon samples from donors 104 and 109, and adherence was quantified as described in Materials and Methods. Panel C shows the results of an adherence experiment with Caco-2 cells. Data represent the mean values of three independent experiments, and the error bars denote the standard error of the mean. Technical replicates, three wells; six biological replicates (two donors and three segments).

Finally, we assessed the total level of adherence of EAEC 042 lacking *aafA* and *aggR* to cultures from two donors, 104 and 109. There was a quantifiable and statistically significant decrease in adherence by EAEC lacking these genes compared to that of wild-type EAEC in the duodenum and ileum of donor 104 (Fig. 5A and B). The low level of colon binding precluded the detection of any difference between the wild-type and mutant strains. There was no difference in the total adherence to any of the segments from donor 109, despite solid levels of adherence by the wild-type strain (Fig. 5A to C), and a detectable and significant decrease in an equivalent experiment performed with intestinal Caco-2 cells.

## DISCUSSION

Bacterially induced diseases are the sum of the virulence factors manifested by the bacterium and the host’s response to the infection. The bacterial side of the equation has principally been investigated; elegant reverse genetics approaches used to examine the function of bacterial genes in infection model systems and structure-function studies of virulence factors have provided insights into the molecular pathogenesis of disease. Often held constant in this equation, however, has been the host, relying principally on multiply passed transformed cell lines (often not from the afflicted tissue) and animal models that may not recapitulate the natural history and pathology of human infection. Whereas this approach has been successful at providing medical solutions to some afflictions caused by bacteria, especially those where there is a strong toxin-based component to the disease (tetanus and diphtheria are good examples), it has fallen short in solving others, particularly with diseases where the host and bacterium can coexist in a chronic associative state.

Infections with EAEC are associated with a diverse range of clinical symptoms, and this heterogeneity complicates control and treatment. These symptoms include watery (suspected small-bowel etiology) or inflammatory (suspected large-bowel etiology) diarrhea and the duration of the infection, which can be transient (a few days) or chronic (weeks) in some pediatric populations. Numerous experimental model systems have been employed to better understand the molecular basis of this heterogeneity. The cell lines used include the HEp-2, T84, and Caco-2 lines. The HEp-2 adherence assay is the “gold standard” for the diagnosis of the stacked-brick phenotype of aggregative adherence. T84 and Caco-2 cells have been used to study putative virulence factors and assess host inflammation (13, 15, 51–53). These are useful systems, but their transformed nature and single-donor status may not represent the breadth of physiology needed to assess this complex disease. Animal models include pigs, rats, mice, and recently rabbits (50). Gnotobiotic pigs may be medically relevant, since pronounced diarrhea is observed in this model, along with villus swelling and edema in the lamina propria (54). Of importance to the results observed here, the stacked-brick pattern of adherence is observed in this model, with an ileal tropism. EAEC bacteria were associated with thick mucus layers, which is notable because human volunteers challenged with EAEC 042 produced mucoid stools, along with a duodenal localization in two of the three challenge subjects, which is consistent with the strong duodenal adherence seen in our study (16). Similar histopathology was observed in a rabbit intestinal loop model, but rabbits succumb to the infection in about half the cases, which is not common in humans (12, 55, 56). Infant rabbits infected with the German Stx-producing hybrid EAEC strain manifest diarrhea (50). Importantly, the diarrhea is dependent on Stx and SPATES but not pAA (encodes fimbriae and AggR); however, the animals do not develop hemolytic-uremic syndrome (HUS), which was observed during the German outbreak. This is notable because the presence of pAA (which encodes the fimbriae and the regulator of adherence AggR) is associated with an increased frequency of HUS and greater disease severity in humans and in culture models is necessary for the translocation of Stx across the intestinal epithelium (57, 58). These findings raise the interesting but untested hypothesis that the presence of adherence fimbriae may exacerbate the disease by bringing EAEC in close proximity to intestinal cells, where toxins and SPATES can exert local cytotoxicity. Nevertheless, the cumbersome and expensive nature of these model systems prevents them from being universally utilized. Neonatal mice have been used to assess the effect of infection on cognitive development (59) and the role of toxins (30), but in general, mice are considered poor surrogates because of a lack of diarrhea and gastrointestinal pathology. Outside human challenge studies, *in vitro* organ culture may be the model system that allows for the most complexity to be assessed; however, the lack of robust access to diverse human tissues and the short time frame of culture (8 h) limit reproducibility (60). Fixed human tissue eliminates the time crunch (61), but the inactivated nature of the tissue prevents assessment of the molecular dynamics of the host-pathogen interaction. Reverse genetic approaches in these systems are essentially nonexistent, except for the mouse, which suffers from a lack of pathology.

Organotypic cultures such as enteroids do not solve all of these limitations, but they do overcome several. In particular, because of their availability and ease of culture, enteroids derived from different donors and across intestinal segments of the same donor allow assessment of the host’s contribution to infection. This revealed stark differences in the adherence pattern and total binding across intestinal segments and donors. Some of the observations are noteworthy, considering previously published reports. There were consistently higher levels of adherence to the duodenum. Previous studies using formalin-fixed tissues either did not assess the duodenum or did not observe it. Our results are consistent with those of Raj et al., who did observe adherence to cultured duodenocytes and in a donor-specific manner (62), and a human challenge study by Nataro et al. that showed a duodenal localization of EAEC (16). The strong adherence to the duodenum was met with equally weak or no adherence to the jejunum. Hicks and Phillips, in two separate studies, did observe adherence to the jejunum, and of the aggregated variety, with some villus pathology (63, 64). Both studies also noted binding to the ileum and colon, with colonocytes showing rounding and enlarged crypt openings. Baldwin and Williams, using *in vitro* organ culture, noted that binding to the colon occurred in aggregates and that these aggregates were seemingly linked by fimbriae, of which four types were classified (65). The pattern of adhered EAEC and the fimbrial extensions were similar to those observed in colonoids in our study. Data presented here suggest that these features were dependent on the major fimbrial subunit, as evidenced by the fact that both the adherence and patterning are lost by mutant strains. We propose that this fimbrial subunit somehow engages a host factor that is differentially expressed, modified, or exposed in the small and large bowels and across different individuals. Since our enteroid monolayers are 100% confluent and maintain their transepithelial resistance throughout the 3-h infection (not shown), it is also likely that this host factor is a component of the cell surface, either secreted or attached. It is intriguing that the addition of human bile completely abolishes every adherence pattern observed in this study, leading, in most cases, to what resembles diffuse adherence (Fig. S6). Thus, in addition to a host-specific factor driving the diverse adherence patterns, there are other physiologically relevant elements (such as bile) that are not built into the enteroid system that alter these phenotypes.

10.1128/mBio.02419-17.6 FIG S6  Effect of human bile acid on aggregative adherence of EAEC. Duodenal (D103), jejunal (J3), and ileal (I11) 2D differentiated HIEMs obtained from different patients were infected with EAEC 042 at an MOI of 10 for 3 h. HIEMs were treated with human bile acids at a concentration of 5% at the time of infection [(+) 5% Bile]. HIEMs without bile acids [(−) Bile)] served as a control. After infection, cells were washed, fixed, stained with Giemsa-Wright stain, and imaged at ×100 to visualize the pattern of bacterial adherence. Technical replicates, three wells representative of 12 images; three biological replicates. Download FIG S6, TIF file, 0.4 MB. Copyright © 2018 Rajan et al. 2018 Rajan et al. This content is distributed under the terms of the Creative Commons Attribution 4.0 International license.

**FIG 6:**
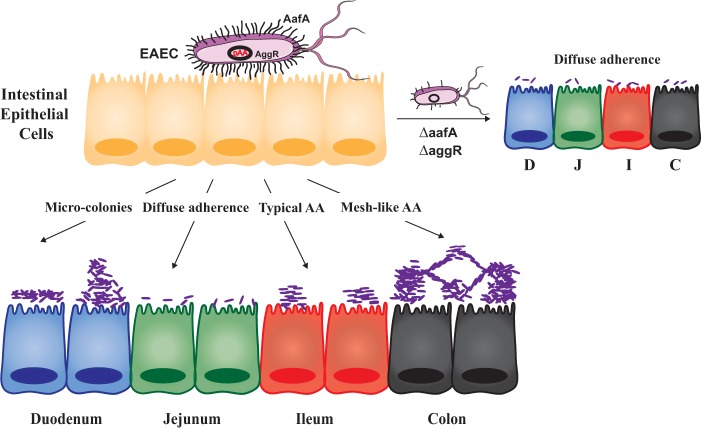
EAEC adherence to human intestinal enteroids. EAEC adheres to intestinal enteroids via five distinct aggregation patterns: sheet-like or microcolony adherence to duodenal (D) cells, diffuse adherence to jejunal (J) cells, typical stacked-brick adherence to ileal (I) cells, and a mesh-like adherence of interconnecting clusters to cells of the colon (C). While these patterns (as well as the strength of the interaction) are dependent on the large subunit of AAF/II, an unidentified host factor mediates adherence across different donors and different intestinal segments.

Human organotypic cultures have the potential to provide novel insights into how enteric pathogens induce diarrhea. They can be grown and infected in 2D and 3D states, which may aid in understanding how tissue architecture affects responses. Enteroids undergo physiological responses such as Na^+^ absorption, Cl^−^ secretion, swelling, and mucin production, all of which are facets of a diarrheal response (43, 45, 66). They can be used to assess novel innate immune responses to pathogens, such as the type III and I interferon responses to rotavirus, and have been used to grow previously unculturable enteric viruses such as norovirus (44, 67). They are currently being used to understand the molecular pathology of *Clostridium difficile* toxin (68, 69), the invasion of *Salmonella enterica* serovar Typhimurium (70), and the effect of Shiga-toxin-producing enterohemorrhagic *E. coli* on the colonic epithelium (45, 60). Gastroids have revealed mechanisms of carcinogenesis by *Helicobacter pylori* (71), and more complex systems are being engineered into such cultures that include immune cells (72), vasculature, and “gut-on-a-chip” approaches with human microbiota (71, 73). Here, human intestinal enteroids revealed a donor- and segment-specific adherence tropism of EAEC, along with several new modes of aggregation (Fig. 6). These modes were dependent on the major fimbrial subunit of EAEC, which seems to bind an unknown host factor. Binding to this factor is not inhibited by mannose or influenced by the secretor status of the individual, as is true for other enteric pathogens (data not shown). Future studies will investigate the identity of this factor and whether it dictates an individual’s susceptibility to EAEC infection.

## MATERIALS AND METHODS

### Bacterial strains.

In this study, we used EAEC 042 (serotype 44:H18), a strain originally isolated from a child in Peru, as the prototype strain of this pathogen (74). Clinical isolates A2A and 5EA were identified by P. Okhuysen; their characteristics are presented in Table 1. HS, a nonpathogenic strain of *E. coli*, was used as a nonaggregative control (75). EAEC 042 strains lacking *aafA* and *aggR* were generously donated by James Nataro. Bacteria were grown overnight in tryptic soy broth at 37°C and subcultured for at least 2 h before every infection. All infections were performed at a multiplicity of infection (MOI) of 10 and a total infection time of 3 h unless otherwise indicated.

### HIEM culture.

Human intestinal enteroid monolayers (HIEMs) were made from 3D enteroid cultures derived from intestinal biopsy specimens from adults undergoing routine endoscopy procedures or bariatric surgeries. All of the biopsy specimens were assessed by physicians, and only the healthy region of intestine was used for enteroid culturing. 3D enteroids were grown in Matrigel (BD Biosciences, San Jose, CA) culture as previously described (37, 40, 43, 48, 66, 67, 76). HIEMs were cultured from 3D enteroids to form monolayers in 96-well plates (for the adherence assay or plating method) or chambered slides (for Giemsa-Wright staining) by a protocol adapted from reference 44. To make HIEMs on chambered slides or coverslips, we coated wells with 2.5 μl of Matrigel diluted in 100 μl of ice-cold phosphate-buffered saline (PBS) and incubated them at 37°C for 20 to 60 min. For HIEMs cultured on 96-well plates, each was coated with 3.3 μl of collagen (Sigma, St. Louis, MO) diluted in 100 μl of water and incubated at 37°C for 90 min. Undifferentiated 3D human intestinal enteroids were washed with 0.5 mM EDTA in ice-cold PBS (no calcium chloride or magnesium chloride), pelleted for 5 min at 1,000 rpm, and dissociated with 0.05% trypsin–0.5 mM EDTA. For trypsin dissociation, enteroids were incubated at 37°C for 4 min for jejunal and colonic segments, 4.5 min for duodenal segments, and 5 min for ileal segments. Trypsin was later inactivated by the addition of complete medium without growth factors [CMGF(−)] and containing 10% fetal bovine serum. Cells were then dissociated to form single-cell suspensions by vigorously pipetting them up and down with a P1000 pipette and passing them through a 40-μm cell strainer. The cells were pelleted for 3.5 min at 1,500 rpm and suspended in 100 μl of complete medium with growth factors [CMGF(+)] containing the ROCK inhibitor Y-27632 (10 μM; Sigma). The cell suspensions were seeded into a single well of 96-well plate or a chambered slide. After 24 h, the culture medium was changed to differentiation medium to allow the differentiation of enterocytes. The cells were then differentiated for 3 to 5 days for all experiments, and the medium was changed every other day. Differentiation medium contains the same components as CMGF(+) medium, with the exception of Wnt3A, SB202190, and nicotinamide. In addition, differentiation medium also has 50% lower concentrations of Noggin and R-spondin. The secretor status of each enteroid line was determined by genotyping as previously described (31, 64). The enteroid cultures were from adults 21 to 69 years old, and the details of the enteroid lines used are shown in Table 2.

**TABLE 2:** Details of the enteroid lines used in this study

Enteroid line^a^	Intestinal segment	Gender^b^	Ethnicity	Sample type
D1	Duodenum	F	African American	Biopsy
D3	Duodenum	M	Caucasian	Biopsy
D4	Duodenum	F	Caucasian	Biopsy
D5	Duodenum	F	Caucasian	Biopsy
D18	Duodenum	M	Hispanic	Biopsy
D103	Duodenum	F	African American	Biopsy
D104	Duodenum	F	Caucasian	Biopsy
D109	Duodenum	F	Hispanic	Biopsy
4D^c^	Duodenum	NA	NA	NA
8D^c^	Duodenum	NA	NA	NA
J3	Jejunum	F	NA	Bariatric
J8	Jejunum	F	NA	Bariatric
J10	Jejunum	NA	NA	Bariatric
J11	Jejunum	F	NA	Bariatric
IL5	Ileum	M	African American	Biopsy
IL11	Ileum	F	African American	Biopsy
IL12	Ileum	F	African American	Biopsy
IL15	Ileum	F	Caucasian	Biopsy
IL16	Ileum	M	Hispanic	Biopsy
I103	Ileum	F	African American	Biopsy
I104	Ileum	F	Caucasian	Biopsy
I109	Ileum	F	Hispanic	Biopsy
CO1	Transverse colon	F	Caucasian	Biopsy
CO2	Ascending colon	F	Asian	Biopsy
CO4	Ascending colon	M	Caucasian	Biopsy
C103	Ascending colon	F	African American	Biopsy
C104	Colon	F	Caucasian	Biopsy
C109	Ascending colon	F	Hispanic	Biopsy

a The letters before the numbers refer to the intestinal segment. The numbers refer to our institutional designation for the donor.

b F, female; M, male; NA, not available.

c Enteroids obtained from pediatric samples (age, <10 years).

### Cell culture.

Caco-2 colonic cells (ATCC CRL-2102) and HEp-2 cells (ATCC CCL-23) were cultured in 75-cm^2^ flasks in Dulbecco’s modified Eagle’s medium (Mediatech, Inc. [a Corning subsidiary], Manassas, VA) supplemented with 10% fetal bovine serum (Atlanta Biologicals, Flowery Branch, GA) and a 1% mixture of 100 U/ml penicillin and 100 μg/ml streptomycin (Thermo Fisher Scientific, Boston, MA). Cells were seeded at a density of 5 × 10^4^/ml at 37°C in the presence of 5% CO_2_ in a humidified incubator.

### Adherence assay.

To measure adherence, HIEMs were differentiated for a minimum of 3 days or up to 5 days. HIEMs were visually checked for the formation of confluent monolayers, and for all experiments, HIEMs were at least 90% confluent. The cells in monolayers were counted as an average of two wells for every experiment performed. To determine the number of cells, medium was removed from the wells and 100 µl of trypsin was added to the monolayer and it was incubated at 37°C in the presence of 5% CO_2_ in a humidified incubator for 5 min. Trypsinization was stopped by transferring cells to a microcentrifuge tube containing 900 µl of CMGF(−) medium and 5% fetal bovine serum. Ten microliters of cell suspension was loaded onto a hemocytometer, and the number of cells in 100 µl was determined in accordance with the manufacturer’s instructions. The number of enteroid cells in the monolayer typically ranged from 50,000 to 100,000. After the number of cells present in monolayers was determined, HIEMs were infected with EAEC suspended in differentiation medium containing no antibiotics at an MOI of 10 and then incubated for 3 h at 37°C in the presence of 5% CO_2_ in a humidified incubator. To measure the adherence of EAEC, monolayers were first washed thrice with PBS and then disrupted by being scraped up and down several times with pipette tips in PBS. The bacterial count was enumerated as follows: Σ Adherent bacteria/well = CFU of adherent bacteria/total number of enteroid cells. This protocol is a modified form of those of Steiner et al. (77) and Vail et al. (78), where HEp-2 cells were used as the model system to test EAEC adherence. MOIs of 0.01, 0.1, 1, 5, 10, 50, and 100 were tested. While the lower MOIs were effective in studying longer time periods, an MOI of >50 at 3 h postinfection resulted in displacement of the enteroid monolayers and host cell death. Thus, an MOI of 10 and a 3-h time point were determined to be optimal for the measurement of adherence. Figure 1A is a schematic of the process.

### Giemsa-Wright staining.

HIEMs cultured on chambered slides and at 4 days of differentiation were infected at an MOI of 10 and incubated for 3 h at 37°C in the presence of 5% CO_2_ in a humidified incubator. The cells were washed three times with PBS to remove any nonadherent bacteria. The cells were then fixed and stained with Hema 3 fixative and solutions (Protocol catalog no. 122-91) and imaged by oil immersion microscopy at ×100 magnification. The aggregative adherence patterns were subtyped on the basis of the following definitions: (i) typical = 2D array of clusters of 15 to 40 bacteria, (ii) microcolonies = 3D array of ≥100 clustered bacteria, iii) sheet-like = 2D array of ≥50 bacteria, and (iv) mesh-like = physically linked clusters of ≥200 bacteria. These patterns were defined per 0.8 mm^2^ of a given field of view for at least 12 different fields (×100 magnification). Diffuse adherence was classified as clusters of only one to four bacteria per field. All assessments were performed by an investigator blinded to the details of the experiment.

### Statistical analysis.

All adherence assay results are the mean and standard error of the mean of triplicate assays performed over several independent enteroid preparations. Statistical significance was determined by two-way analysis of variance. Tukey’s multiple-comparison tests were performed with GraphPad Prism version 7.0 for Windows (Graph Pad Software, San Diego, CA). Differences between mean values were considered significant at *P* ≤ 0.05. Specific *P* values are shown in the figure legends.
