# Supplementary figures and images for: Novel Segment- and Host-Specific Patterns of Enteroaggregative Escherichia coli Adherence to Human Intestinal Enteroids
Source: mBio. 2018 Feb 20;9(1):e02419-17. doi: 10.1128/mBio.02419-17 (PMC5821088; doi:10.1128/mBio.02419-17)

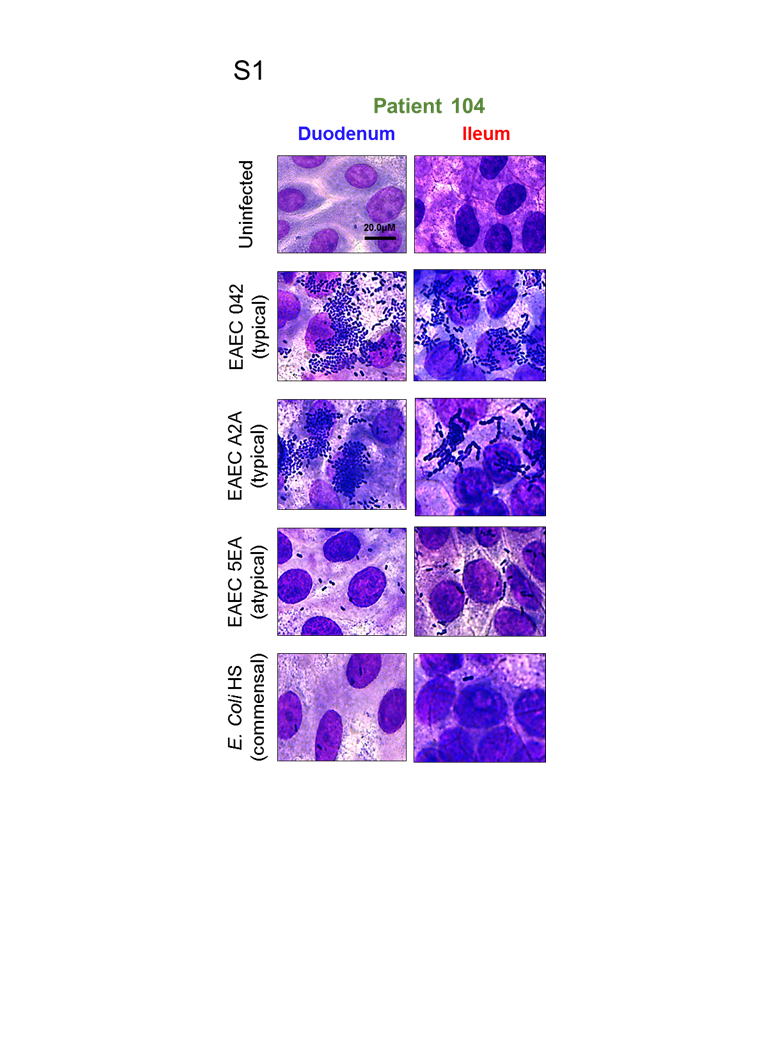

Supplement: FIG S1 [file mbo001183735sf1.tif]

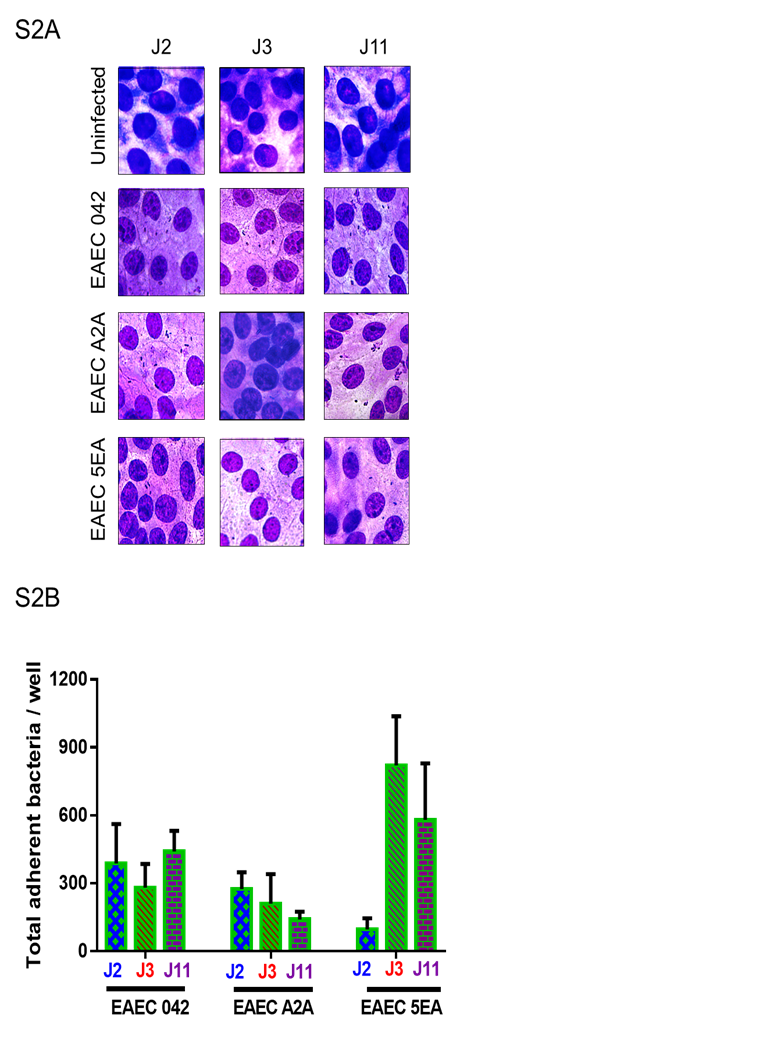

Supplement: FIG S2 [file mbo001183735sf2.tif]

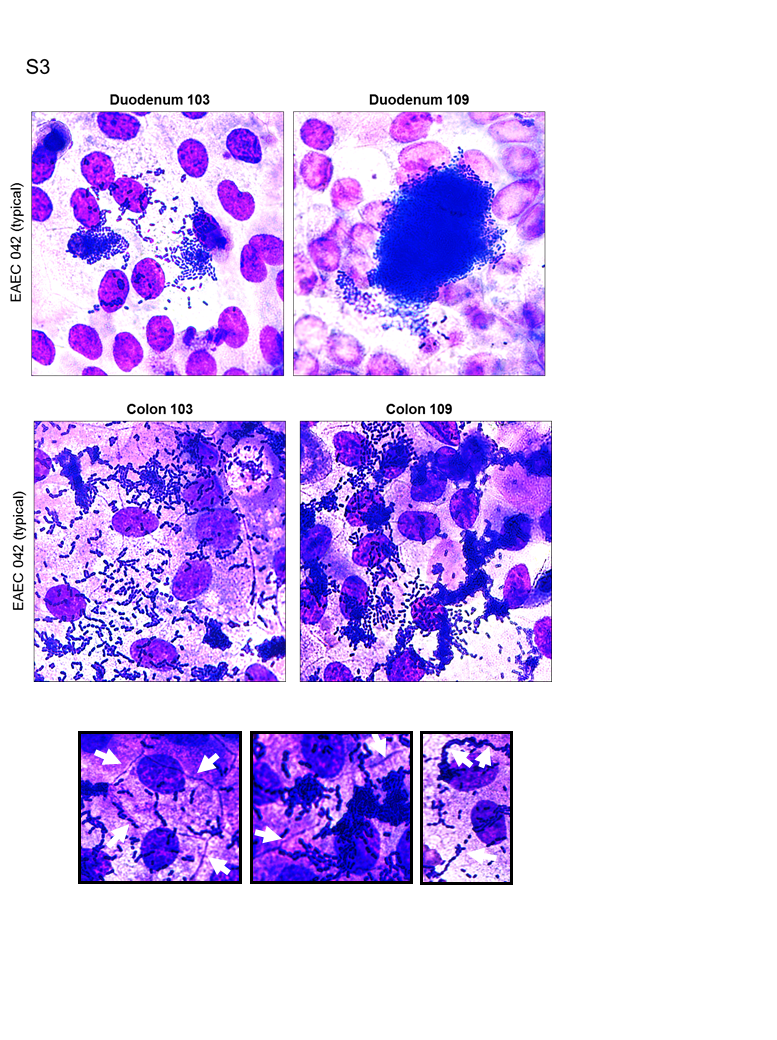

Supplement: FIG S3 [file mbo001183735sf3.tif]

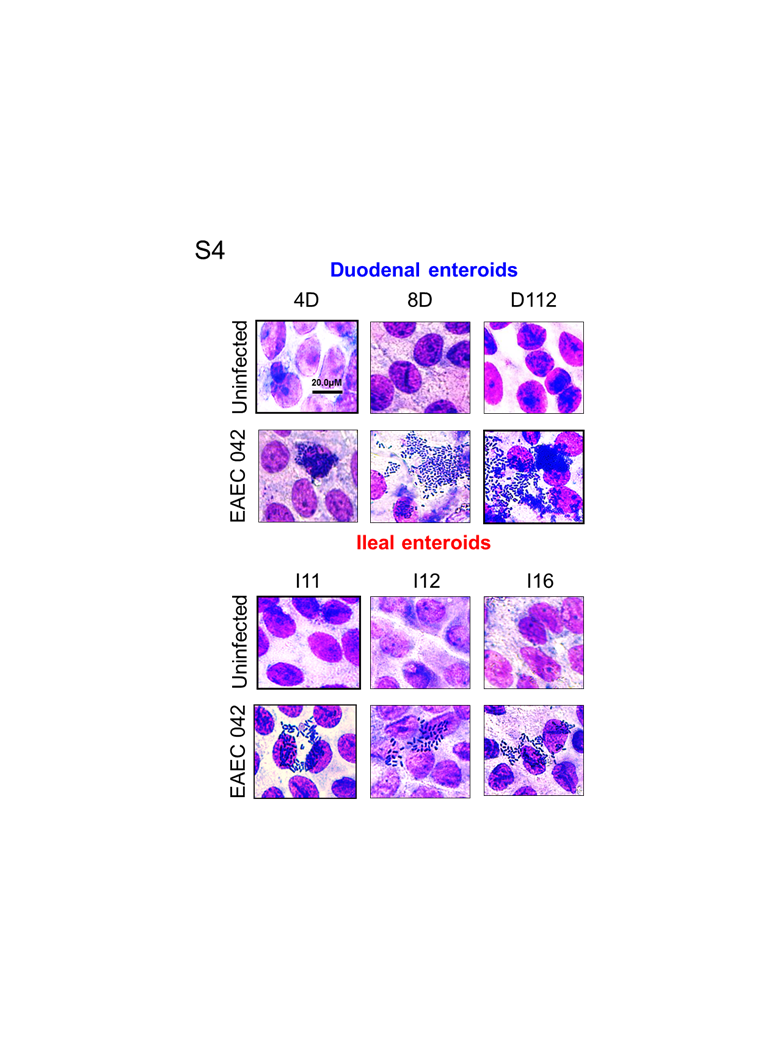

Supplement: FIG S4 [file mbo001183735sf4.tif]

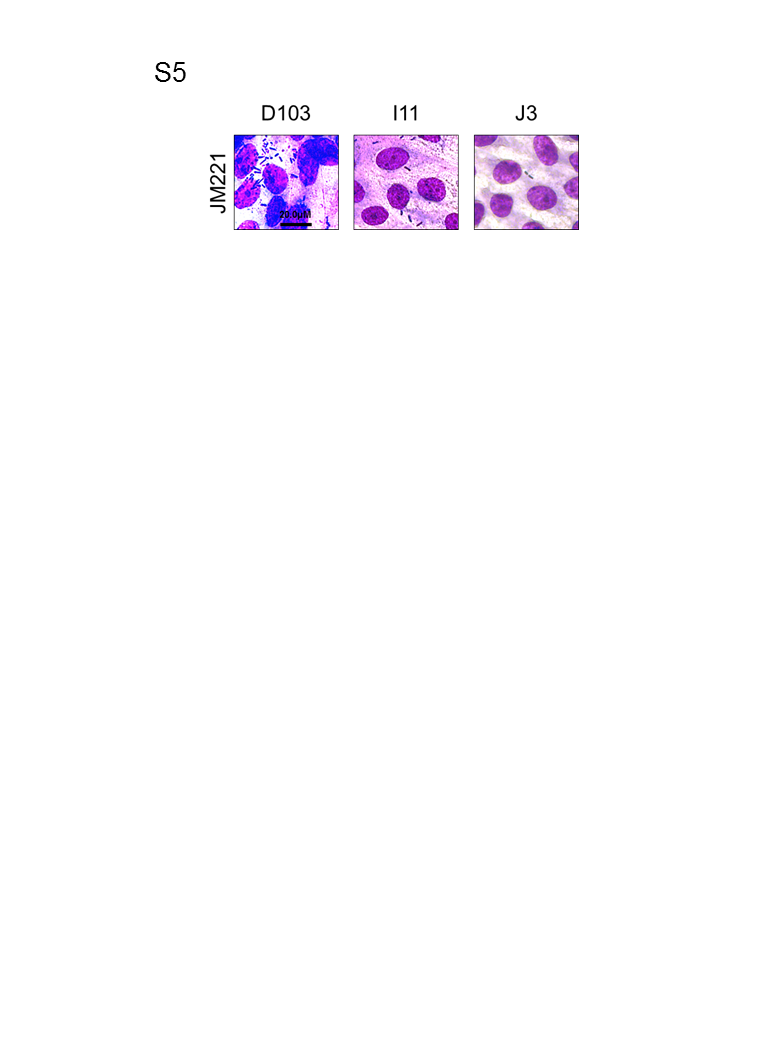

Supplement: FIG S5 [file mbo001183735sf5.tif]

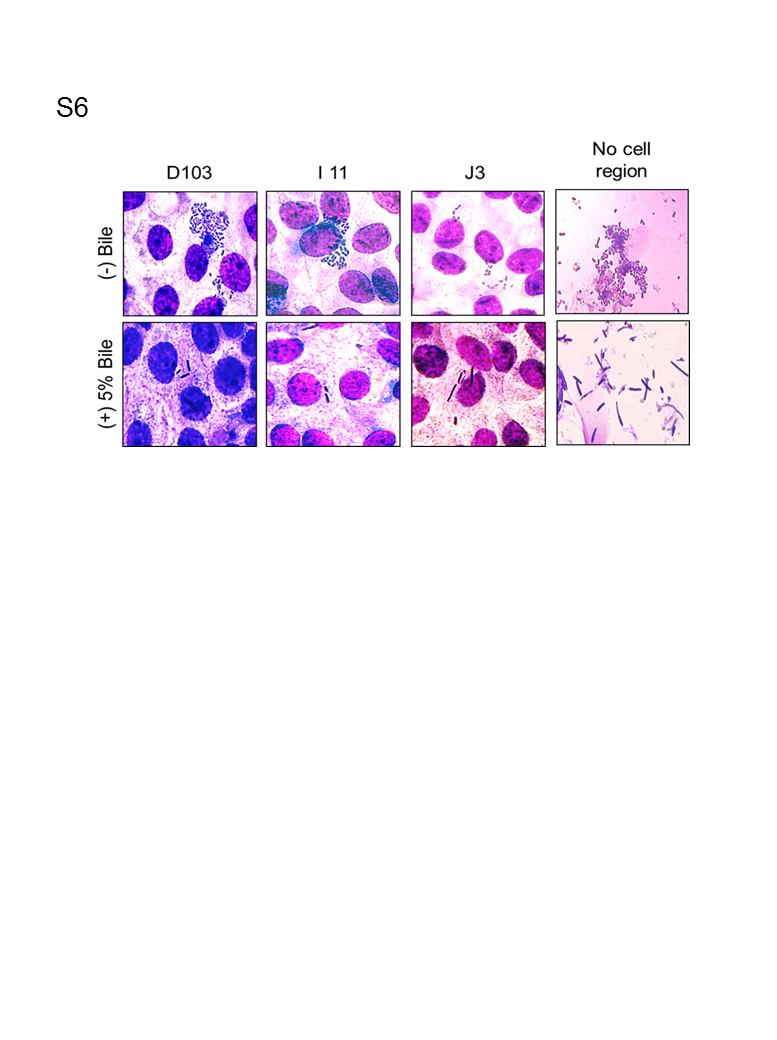

Supplement: FIG S6 [file mbo001183735sf6.tif]
